# Integration of Vibro-Acoustography Imaging Modality with the Traditional Mammography

**DOI:** 10.1155/2007/40980

**Published:** 2007-02-13

**Authors:** H. Gholam Hosseini, A. Alizad, M. Fatemi

**Affiliations:** ^1^School of Engineering, Auckland University of Technology, Private Bag 92006, Auckland 1142, New Zealand; ^2^Deptartment of Physiology and Biomedical Engineering, Mayo Clinic College of Medicine, Rochester, MN 55905, USA

## Abstract

Vibro-acoustography (VA) is a new imaging modality that has been applied to both medical and industrial imaging. Integrating unique diagnostic information of VA with other medical imaging is one of our research interests. In this work, we establish correspondence between the VA images and traditional X-ray mammogram by adopting a flexible control-point selection technique for image registration. A modified second-order polynomial, which simply leads to a scale/rotation/translation invariant registration, was used. The results of registration were used to spatially transform the breast VA images to map with the X-ray mammography with a registration error of less than 1.65 mm. The fused image is defined as a linear integration of the VA and X-ray images. Moreover, a color-based fusion technique was employed to integrate the images for better visualization of structural information.

## 1. INTRODUCTION

Screening X-ray mammography is continue to be the primary tool for early detection of breast cancer and therefore reduces the mortality rate of the disease. Over the past few years, the quality of mammography has improved significantly but the accuracy of image interpretation is still a remained challenge. Mammography interpretation depends on human factors which is difficult to quantify. Thus, ensuring accurate interpretation of mammography is important for women's health [1].

Vibro-acoustography (VA) is a new imaging modality based on ultrasound-stimulated acoustic emission which can be integrated with the mammography to enhance breast cancer diagnosis. The VA acoustic field in response to vibration of an object due to an applied cyclic force at each point is detected by a hydrophone and used to form the image of the object [2].

Vibro-acoustography has been tested as a noninvasive imaging tool to image excised human tissues, such as breast [3], liver [4], arteries [5], and prostate [6]. Vibro-acoustography has also been used as a nondestructive imaging tool to identify the structural flaws of materials by measuring changes in the mechanical response to vibration at a point of interest [7, 8].

Recently, we have developed a VA system for in vivo breast imaging [3]. This system is integrated with a clinical stereotactic mammogram machine. The combined system is designed to produce matching VA and mammography images of the breast. The dual modality system can serve two purposes. The mammogram is used as a reference image to evaluate and optimize VA performance. Secondly, it is anticipated that the VA and mammography images would provide complimentary information of the breast. Thus, by integrating the two images, the diagnostic value of the two-modality image would be more than the individual images.

While mammography is considered as an important diagnostic tool, particularly for screening microcalcification clusters and detecting malignancy [9–11], there are associated shortcomings which raise concern about the quality of image interpretation. For example, the efficacy of this modality heavily decreases in dense breast imaging [12]. Moreover, X-ray mammography does not contain information about the depth and thickness of the objects. Vibro-acoustography, on the other hand, is not hampered by tissue density [3].

The above argument further justifies integrating VA and mammography. Integration of multimodality imaging has been widely used for generating more diagnostic and clinical values in medical imaging. Proper image registration and multimodality image fusion techniques need to be employed for high quality image integration. On the other hand, inaccurate image registration and incorrect localization of region of interest risks a potential impact on patients. Integrating images of the same target generated with different modalities has been investigated for various clinical images [13–15].

In a study published by Behrenbruch et al. [13], fusion of the high-resolution structural information available from mammography with the functional data acquired from MRI imaging is proposed to offer a better pathological indicator such as calcifications. It has been reported that some tissue details that are not visible in contrast-enhanced MRI can be recognized in the fused images [13].

Prior to the fusion process, it is important to apply a robust registration technique to align images, from a single or from different modalities [14, 15]. By image registration, the correspondences between the images can be seen more easily and the clinicians can get maximum amount of information from the images [15].

This paper describes a reliable image registration technique for aligning VA images and X-ray mammogram. It also proposes principles of integrating VA and X-ray images after performing a reliable registration. As a result, a software-based image alignment tool was designed for integrating the two modalities and facilitating the diagnosis process. Assuming that these two completely different modalities should provide relatively independent information about the breast tissues, the ultimate aim of this research is to enhance the quality of image interpretation and further improving the effectiveness of breast cancer detection.

## 2. METHODS AND MATERIALS

### 2.1. Experiment setup

**Figure 1:**
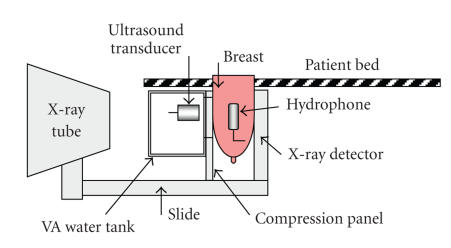
Schematic of combined vibro-acoustography-mammography system, “reproduced with permission from [3].”

Figure 1 shows a schematic of combined vibro-acoustography-mammography system used for image generation. X-ray images are generated by Mammotest/Mammovision (Fischer Imaging Corporation's HF-X Mammography) system equipped with compression paddle to immobilize the target (breast). Vibro-acoustography transducer is mounted in a water tank attached to the mammography system (see Figure 1). This transducer is designed with two arrays (two compact transducers) driven by two continuous-wave or tone-burst signals at slightly different frequencies [16]. A window (104-by-80 mm) covered by a flexible membrane is mounted on water tank wall to allow both X-ray and the ultrasound beams to pass through to the target. The patient breast is covered by ultrasound coupling gel before it is placed in contact with the membrane. The imaging window for either imaging method is a 50-by-50 mm square. Within this area, the VA collects 256-by-256 points of the target by scanning the breast.

### 2.2. Principles of vibro-acoustography

Vibro-acoustography is based on low-frequency vibrations induced in the object due to the radiation force of ultrasound. Radiation force is generated by a change in the spatial distribution of the energy density of an incident ultrasound beam. The change of energy density of the impinging ultrasound may be due to energy absorption, scattering, and reflection.

The magnitude of radiation force depends on a number of parameters, including the scattering and absorption properties of the object. For a planar object insonified with a plane wave, the radiation force is related to the power reflection coefficient of the object [3, 7].

In vibro-acoustography, two continuous wave (CW) ultrasound beams of slightly different frequencies, *f* 0 = 3 MHz and *f* 0 + Δ*f*, are used with Δ*f* = 30 KHz. The two beams are focused at a joint focal point. At this point, the combined ultrasound field energy density is sinusoidally modulated. Therefore, the field generates a highly localized oscillatory radiation force at frequency Δ*f*, when interacting with an object. The radiation stress is normally confined to a small volume of the object, which acts as an oscillating point force placed remotely inside the object [3].

The radiation force vibrates the object at frequency Δ*f* and generates in a secondary acoustic field (acoustic emission) with the same frequency that propagates in the object. As the ultrasound beam is scanned across the object, an audio hydrophone can detect the acoustic emission. The hydrophone signal is recorded and its amplified amplitude is mapped into an image [3, 7].

Other VA system parameters are: the resolution of the VA setup = 0.7 mm in transverse plane and about 9 mm in axial direction, spatial sampling interval is 0.2 mm in both directions in the scan plane, the scan time is about 7 minutes for a 256 × 256 image, and ultrasound intensity at the focal point = 700 mW/cm^2^ in compliance with the FDA recommendation for in vivo ultrasound. The audio hydrophone is covered with acoustic gel and placed in contact with the side of breast. The ultrasound transducer can scan the breast through a window. To take an X-ray, the water tank is emptied and the transducer is moved out of X-ray path [5].

The vibro-acoustogram illustrates two types of information about the object: (i) ultrasonic properties of the object, such as the scattering and power absorption characteristics; (ii) the dynamic characteristics of the object at frequency Δ*f*, which also relates to the boundary conditions and the coupling to the surrounding medium. The acoustic emission is also influenced by the surrounding medium as it propagates from the focal point of the transducer to the hydrophone. As the transducer scans the object, the distance between the focal point and the hydrophone varies. However, because the attenuation of the tissue at Δ*f* is not significant, variations of the acoustic emission due to variations of focal point-to-hydrophone distance is negligible. The ultrasonic properties are those that are also present in conventional ultrasound imaging. The dynamic characteristics at Δ*f*, which are related to object stiffness, can be described in terms of object mechanical impedance at frequency Δ*f*. Such information is not available from conventional ultrasonography [3].

Speckle is the snowy pattern which results from random interference of the scattered ultrasound field. Speckles reduce the contrasts of conventional ultrasound images and often limit detection of small structures, such as breast micro calcifications in tissue. Vibro-acoustography on the other hand uses the acoustic emission signal, which is at a low frequency, thus the resulting images are speckle free and have high contrast. This feature makes vibro-acoustography suitable for detection of breast micro calcifications [3].

### 2.3. Theory of image registration

**Figure 2:**
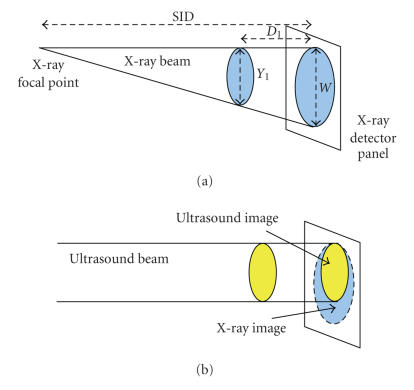
Schematic of beams and target coverage areas in (a) X-ray; (b) VA.

The VA beams stay parallel as the object is scanned and can be used to generate 3D images of an object by integrating its image slices acquired at different depths. The VA beams scan across the object while focusing at a fixed depth. Various slices of the object are scanned at different depths and the corresponding VA images are formed. On the other hand, X-ray beams form a perspective projection image and generate a 2D image of the object without any depth/thickness information. Consequently, the VA images have a fixed magnification for targets at different depths but the X-ray images show variable magnifications depending on target depth. Figure 2 shows a schematic of beams and target coverage areas (filled ovals) in these two modalities. It is shown, in Figure 2(b), that the screened images of X-ray (large oval) and VA (small oval) of the same target are different. Therefore, for the purpose of overlaying the images, a geometric calibration is required to resize and shift one image to match with the other one.

Ideally, by registration, the size and position of VA images should be geometrically transformed to match exactly with mammography images pixel-by-pixel. In this work, we adopted an algorithm based on control points (CPs) and found an equation to adjust the registration transformation with a magnification factor (MF) for different depths. CPs can be identified in an image as pixel points related to user added markers or existing image spots [17]. The MF is defined by the ratio of *W* and *Y*_1_ where *W* is the dimension of the image at the plane of the X-ray detector and *Y*_1_ is the dimension of the target (see Figure 2(a)) by X-ray. Equation (1) expresses MF as the ratio of *W* and *Y*_1_ and also versus SID and *D*_1_ as follows:

(1) $$\text{MF}=\frac{W}{Y_{1}}=\frac{\text{SID}}{\text{SID}-D_{1}}$$,

where SID is equal to 664 mm and *D*_1_ is the distance of the target from the X-ray detector panel.

In Mammovision system, SID is defined as “source to image” or “focal spot to image receptor” distance which is equal to 664 mm in this case. For VA, the focal point is located at 70 mm distance from the transducer. The position of the transducer is changeable to focus at different depths in the object (target). As an example, any target located within a range of 10 mm to 100 mm distance from the X-ray detector panel can be scanned.

The sensitive part of the X-ray detector panel is a 50 × 50 mm square CCD screen. The stereotactic mammography system has target-focusing interface that can be used for measuring *D*_1_. This system establishes depth of the target using stereo images taken by positioning the X-ray camera at +15 and −15 degree deviation related to its normal zeroth-degree position. The dept information is important in the understanding of the calibration system and error propagation.

**Figure 3:**
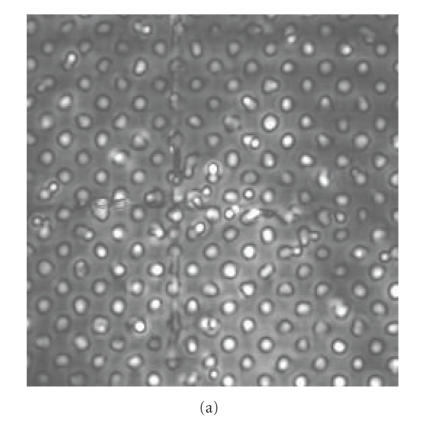

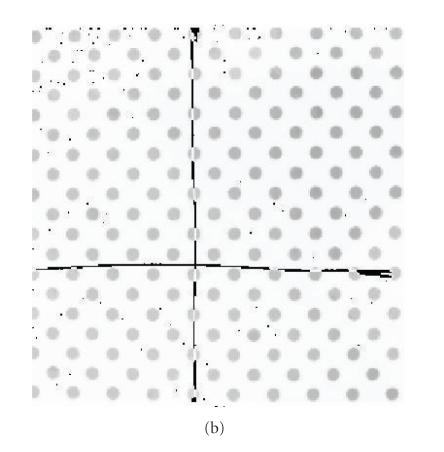
Images of the phantom used to create the matrix of control points: (a) VA image; (b) X-ray image.

An initial study of X-ray and VA images of a phantom was conducted to create a universal matrix of CPs. This matrix can be used in registering the breast images for the purpose of integrating two modalities. The phantom was fabricated from a perforated PVC board with two cross wires mounted as markers. It is used to mimic 2D targets and validate the registration method by calculating the target registration error (TRE) [18, 19]. The images acquired from the same phantom by VA and X-ray techniques are illustrated in Figures 3(a) and 3(b), respectively.

The CPs are selected along the image of two cross wires to ensure that the same points were selected in the X-ray and VA image. We used acoustic gel to couple the hydrophone to the PVC board to reduce bubbles which appear as white points in the VA images. The mid grey points on Figure 3(b) are part of the X-ray image and can be considered as pepper noise. The X-ray images are rescaled from resolution of 1024-by-1024 to 256-by-256 to match the number of pixels with the VA pixels. However, the positions of similar objects (such as CPs) are still different in the two images.

**Figure 4:**
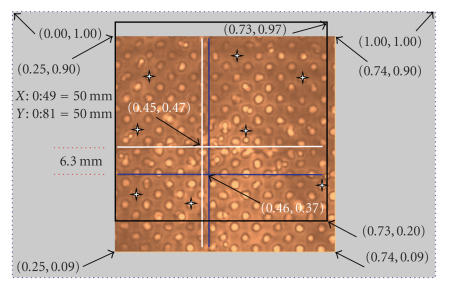
The normalized positions of similar points and the distance between two crossed lines in VA and X-ray images.

Figure 4 shows the normalized positions of similar points and the distance between two cross lines in two images. By assigning (1.00, 1.00) to the position of the very top-right pixel (256, 256) in Figure 4, we can normalize every pixel position of the image.

To establish correspondence between the phantom image obtained by VA with its X-ray image, we plotted a “black square” to illustrate the position of the X-ray frame (in Figure 4). Two cross wires mounted as markers are plotted by white cross lines for the VA image and blue lines for the corresponding X-ray. Figure 4 shows the overlap between the coverage areas of each modality. It shows that by repositioning the VA transducer, we can maximize the common coverage area by two modalities. In addition, due to different magnifications of these two methods still registration is required.

The CPs in X-ray image are selected as base points and similar points in the corresponding VA image are called input points. Eight CPs that can be clearly located in both images were selected to generate two 8-by-2 matrices of base points and input points. The number of CPs and their locations are flexible and can be optimized empirically. The locations of CPs which are the center of eight selected holes in the phantom are indicated by 4-point stars in Figure 4. These matrices are contained the *X* and *Y* coordinates of the selected CPs in the X-ray and the corresponding VA images.

One of the key components of the registration process is to find a mathematical transformation to map the input image to the base image. A second-order polynomial, which is invariant to rotation and translation, was used to infer a spatial transformation of the *X* and *Y* pair of each pixel. For VA registration, this transformation can be applied to the base and input points to map any new grayscale VA image into its corresponding X-ray. The rotation and translation of VA images are mathematically assigned by the transformation, which is then used to create the fusion display of the original grayscale medical images.

The second-order polynomial transformation maps *X*_*b*_ and *Y*_*b*_ of the base-point matrix to *X*_*i*_ and *Y*_*i*_ of the input-points matrix according to (2) [20]:

(2) [*X_i_*, *Y_i_*] = [1,*X_b_*,*Y_b_*,*X_b_* ∗ *Y_b_*,$$X_{b}^{2},Y_{b}^{2}$$] ∗ Inv *T*,

where ∗ is the multiplication sign.

To specify all coefficients of Inv *T* with the size of 6-by-2, at least 6 CPs are required to solve the inverse of the second-order polynomial, Inv *T* [20].

We chose eight CPs and used normalized cross-correlation to adjust each pair of CPs to solve the second-order polynomial.

To adjust the X-ray image for different depths, it is scaled up by MF before applying the transformation of (2). Two-dimensional interpolation techniques such as nearest-neighbor, bilinear, and bicubic interpolation can be used to estimate the image value at a location in between image pixels.

Inaccurate image registration of VA and mammogram may cause incorrect localization of region of interest and may have a potential impact on interpretation of diagnostic information [18]. It is possible to find an adaptive transformation to correct the magnification problem and limit the TRE. This is a suggestion for future research.

### 2.4. Registration of VA breast images and X-ray mammography

**Figure 5:**
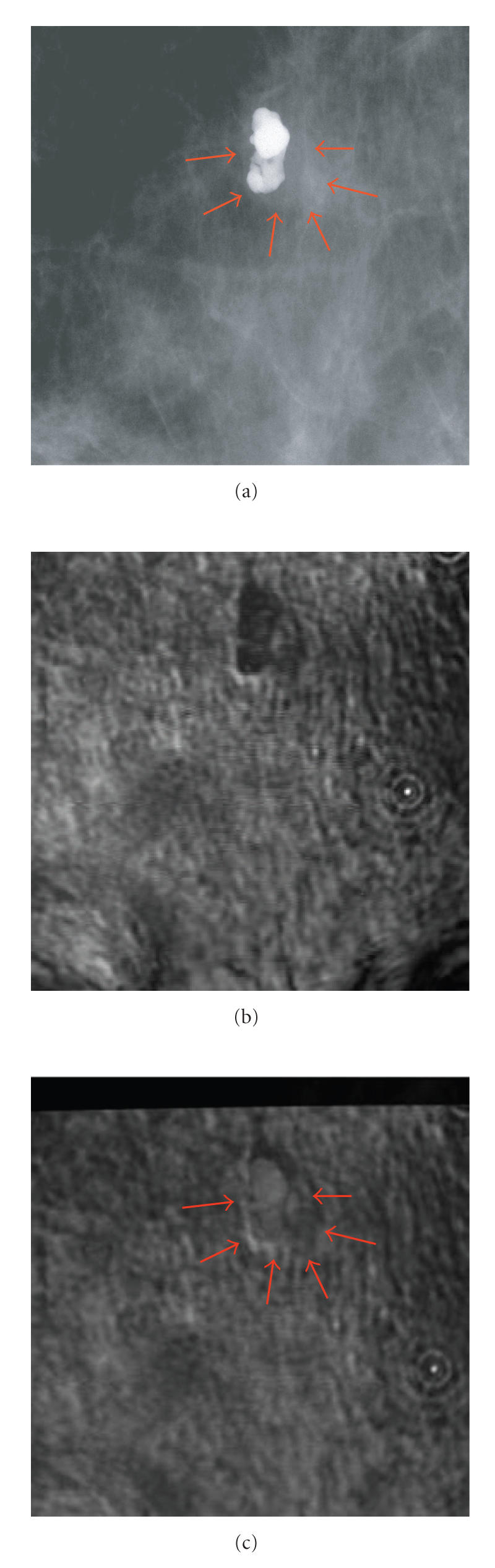
The breast image with fibroadenoma: (a) marked X-ray by an expert radiologist showing the fibroadenoma and the nearby calcification; (b) a 5 × 5 cm^2^ VA image of a breast including a large calcification and nearby fibroadenoma; (c) the resultant registered VA images with focal point at 20 mm from the CCD screen, “reproduced with permission from [3].”

The results of registration of the phantom images including the matrices of base points and input points were used to spatially transform in vivo breast VA images from volunteer. Figure 5(a) shows the coronal view X-ray mammography of the breast of a patient with a large calcification enclosed in a fibroadenoma region marked with arrows according to a radiologist diagnosis. The VA breast image obtained by scanning the same subject with focal point positioned at 4 cm from the skin (20 mm from the CCD screen) is shown in Figure 5(b).

To map the VA image pixel-by-pixel on to the mammogram, a modified polynomial transformation was applied. The resultant registered image is shown in Figure 5(c). We imported the arrows from Figure 5(a) to this figure, at positions that matched the arrows in X-ray image to show the corresponding marking area in the X-ray image. The thick dark band at the top of registered VA image (see Figure 5(c)) is the area that was not covered by VA because the VA image frame did not match exactly. On the other hand, the bottom part of the target, which was scanned by VA, was not covered by X-ray. This error is caused due to misalignment of the X-ray and VA imaging windows. Figure 4 shows clearly the coverage area of each method. However, the rest of VA image is registered to match with its corresponding X-ray image.

### 2.5. Registration error

To evaluate the registration error, three different measures of registration accuracy can be defined as follows [21].

- Fiducial registration error (FRE), which is the value of the point-based registration cost function after registration.
- Surface registration error (SRE), which is the value of the surface-based registration cost function.
- Target registration error (TRE), which is defined as the distance between corresponding points other than those used to estimate the transformation parameters.

The TRE is a more objective measure of registration accuracy but it is difficult to quantify this error in an unmarked registered image. To measure this error, one marker needs to be assigned randomly on each patient as a target and four other markers as fiducials [21].

However, the FRE is a better parameter to measure the registration error in this study as no marker put on the patient. It measures the residual displacement between the points used for registration as the RMS (root mean square) error on the distance between the corresponding CPs of the registered VA and X-ray images,

(3) $$\text{FRE}=\frac{\sqrt{{\sum_{n=1}^{N}d}_{n}^{2}}}{\mathrm{N,}}$$

where *N* is the total number of CPs and *d*_*n*_ is the minimum Euclidean distance between the *n*th CPs. The Euclidean distance, *d*, is the minimum distance between a base-point (*X*_*b*_, *Y*_*b*_) and input-point (*X*_*i*_ and *Y*_*i*_) [22]:

(4) $$d=\min\left\{\sqrt{{\left(X_{b}-X_{i}\right)}^{2}+{\left(Y_{b}-Y_{i}\right)}^{2}}\right\}$$.

The FRE is an accumulation of different error components, such as MF, error due to the registration transformation, and error due to different characteristics of imaging techniques. It was found that the MF is the most dominate component of FRE. To minimize this error, we obtained matrices of CPs for the target located at *D*_1_ equal to 20 mm, which is approximately the mean distance from the CCD screen to the middle of the breast. It is assumed that the maximum size of the compressed breast is less than 40 mm. Therefore, the MF, as expressed by (1), can be modified as follows:

(5) MF_*m*_ = (MF−0.0311).

MF_*m*_ is equal to one for the average distance (*D*_1_ = 20 mm) and (MF = 1.0311). The results of MF_*m*_ calculations for the minimum (*D*_1_ = 0 mm), average (*D*_1_ = 20 mm), and maximum (*D*_1_ = 40 mm) distance of the target are shown in Table 1. By selecting fixed CPs matrices for all target locations, the maximum error due to MF is 1.65 mm ((1.033 − 1) × 50 = 1.65 mm) for a 50-by-50 mm^2^ object.

### 2.6. Image fusion

**Figure 6:**
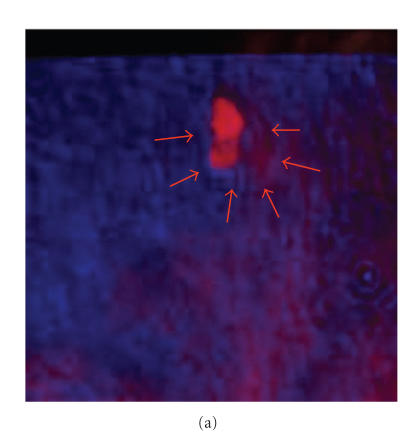

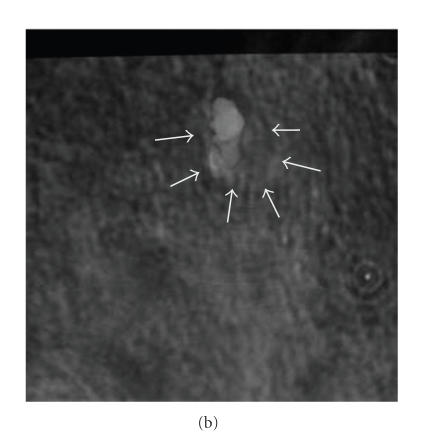
The fused breast image using the primary breast image with fibroadenoma as shown in Figure 5 by (a) color-based method; (b) pixel-level method with R = 50%.

Image fusion can be performed at three different levels: pixel level, feature level, and decision level [23]. We used pixel level image fusion techniques and for better visualization of the structural information contained in both images, it is decided to adopt a color-based method for fusing the registered images. The registered VA and X-ray images are assigned as the blue and red components of an RGB image, respectively. A zero matrix is assigned as the green-component of the RGB image. Figure 6(a) shows the resultant image of color-based fusion of the two primary images shown in Figure 5. This method generates an image with color-code information of each image which may be useful for diagnostic purposes.

To improve the quality of fused images, we used pixel level fusion with different ratio (R) of pixel values. Figure 6(b) shows the resultant image by fusing the two images using 50% of each image pixel value. The integrated image shows features of VA and X-ray in a single image. The calcification seen in the VA and X-ray mammography matches perfectly.

The proposed method for integrating of multimodality medical images allows extracting new information by fusing VA images at different depths with X-ray mammogram. User can select one VA image at a time from a file, containing VA images scanned at different depths of the object, and register it with a based mammogram. Finally, the registered VA image can be enhanced and fused with the base image of X-ray mammography.

**Figure 7:**
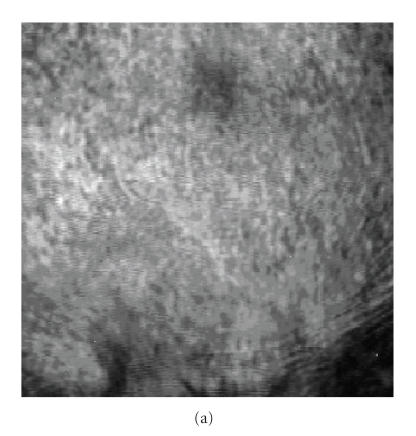

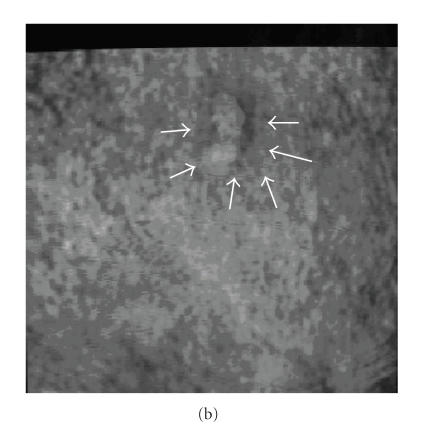
(a) The original VA image; (b) the fusion of VA with marked mammogram.

Figure 7(a) shows another scanned VA image taken at the depth of 5 mm of the CCD screen and Figure 7(b) shows the same image after fusing with the X-ray image which confirms the position of calcification. The VA image shows the fibroadenoma (marked by arrows) which is not clearly visible in the X-ray.

## 3. DISCUSSION AND CONCLUSIONS

Integrating images from two completely different modalities, VA and X-ray, using either color-based or pixel-value fusion techniques may generate more structural and diagnostic information. A color-based fusion technique may be more suitable for visualization of the structural information.

Here, we presented a method for integrating VA and X-ray (mammography). It is shown that, because X-ray image magnification varies with target depth, the registration transformation must be adjusted with a magnification factor for different target depths. In this work, we used a modified second-order polynomial, which leads to a scale/rotation/translation invariant paradigm for image registration. To validate the proposed registration method, we fused VA images at different depths with the X-ray mammogram and demonstrated that the detected classification area is located at the same position in both modalities.

In addition, a method of calculating the fiducial registration error (FRE) due to MF was proposed. By selecting the matrices of CPs for the target located at mid distance from the screen, the error of image registration can be limited to 1.65 mm. In most cases, the size of target is larger than the maximum error and it is positioned at around 20 mm from the CCD screen. Therefore, the registration error is not significant for such targets. We also note that the FRE of 1.65 is about twice the lateral resolution of the system (0.7 mm), indicating the possibility of one resolution cell error in image registration. However, for registering of small targets such as micro calcification, an adaptive transformation can be applied to reduce the FRE.

The fused image of two different modalities which is associated with an X-ray mammography, annotated by an expert radiologist, can be used to verify independent diagnostic information of the VA modality. Moreover, the aligned image would assist the users to gain maximum amount of information from X-ray mammogram and the VA modality.

## Figures and Tables

**Table 1:** Results of MF_*M*_ calculations for different *D*_1_.

*D*_1_(mm)	MF	MF_*m*_

0	1	0.9689
20	1.0311	1
40	1.0641	1.0330

## References

[1] Nass S, Ball J (2005). Improving breast imaging quality standards. *Committee on Improving Mammography Quality Standards*.

[2] Fatemi M, Greenleaf JF (1999). Vibro-acoustography: an imaging modality based on ultrasound-stimulated acoustic emission. *Proceedings of the National Academy of Sciences of the United States of America*.

[3] Alizad A, Whaley DH, Greenleaf JF, Fatemi M (2005). Potential applications of vibro-acoustography in breast imaging. *Technology in Cancer Research and Treatment*.

[4] Alizad A, Wold LE, Greenleaf JF, Fatemi M (2004). Imaging mass lesions by vibro-acoustography: modeling and experiments. *IEEE Transactions on Medical Imaging*.

[5] Alizad A, Fatemi M, Whaley DH, Greenleaf JF (2004). Application of vibro-acoustography for detection of calcified arteries in breast tissue. *Journal of Ultrasound in Medicine*.

[6] Fatemi M, Manduca A, Greenleaf JF (2003). Imaging elastic properties of biological tissues by low-frequency harmonic vibration. *Proceedings of the IEEE*.

[7] Fatemi M, Wold LE, Alizad A, Greenleaf JF (2002). Vibro-acoustic tissue mammography. *IEEE Transactions on Medical Imaging*.

[8] Fatemi M, Greenleaf JF (1998). Ultrasound-stimulated vibro-acoustic spectrography. *Science*.

[9] Hendee WR (1995). History and status of x-ray mammography. *Health Physics*.

[10] Highnam R, Brady M (1999). *Mammographic Image Analysis*.

[11] Aichinger H, Dierker J, Joite-Barfuss S, Sabel M (2004). Radiation exposure and image quality in x-ray diagnostic radiology. *Physical Principles and Clinical Applications*.

[12] Alizad A, Fatemi M, Wold LE, Greenleaf JF (2004). Performance of vibro-acoustography in detecting microcalcifications in excised human breast tissue: a study of 74 tissue samples. *IEEE Transactions on Medical Imaging*.

[13] Behrenbruch CP, Marias K, Armitage PA (2003). Fusion of contrast-enhanced breast MR and mammographic imaging data. *Medical Image Analysis*.

[14] Kapoor R, Dutta A, Bagai D, Kamal TS Fusion for registration of medical images—a study.

[15] Hallpike L, Hawkes DJ (2002). Medical image registration: an overview. *Imaging*.

[16] Silva GT, Greenleaf JF, Fatemi M (2004). Linear arrays for vibro-acoustography: a numerical simulation study. *Ultrasonic Imaging*.

[17] Li W, Leung H (2004). A maximum likelihood approach for image registration using control point and intensity. *IEEE Transactions on Image Processing*.

[18] Porter BC, Rubens DJ, Strang JG, Smith J, Totterman S, Parker KJ (2001). Three-dimensional registration and fusion of ultrasound and MRI using major vessels as fiducial markers. *IEEE Transactions on Medical Imaging*.

[19] Hawkes DJ, Hajnal JV, Hill DLG, Hawkes DJ (2001). Registration methodology: introduction. *Medical Image Registration*.

[20] Goshtasby A (1988). Image registration by local approximation methods. *Image and Vision Computing*.

[21] Maurer CR, Aboutanos GB, Dawant BM, Maciunas RJ, Fitzpatrick JM (1996). Registration of 3-D images using weighted geometrical features. *IEEE Transactions on Medical Imaging*.

[22] Paragios N, Rousson M, Ramesh V (2003). Non-rigid registration using distance functions. *Computer Vision and Image Understanding*.

[23] Kapur A, Carson PL, Eberhard J (2004). Combination of digital mammography with semi-automated 3D breast ultrasound. *Technology in Cancer Research and Treatment*.

